# Macrophage migration inhibitory factor regulates interleukin-6 production by facilitating nuclear factor-kappa B activation during *Vibrio vulnificus *infection

**DOI:** 10.1186/1471-2172-11-50

**Published:** 2010-10-12

**Authors:** Chia-Chang Chuang, Yin-Ching Chuang, Wen-Teng Chang, Chi-Chung Chen, Lien-I Hor, A-Ming Huang, Pui-Ching Choi, Chi-Yun Wang, Po-Chin Tseng, Chiou-Feng Lin

**Affiliations:** 1 Institute of Clinical Medicine, College of Medicine, National Cheng Kung University, Tainan 701, Taiwan; 2 Department of Emergency Medicine, National Cheng Kung University Hospital, Tainan 701, Taiwan; 3 Department of Medical Research, Chi Mei Medical Center, Tainan County 710, Taiwan; 4 Department of Biological Science and Technology, Chung Hwa University of Medical Technology, Tainan County 717, Taiwan; 5 Department of Physiology, College of Medicine, National Cheng Kung University, Tainan 701, Taiwan; 6 Department of Microbiology and Immunology, College of Medicine, National Cheng Kung University, Tainan 701, Taiwan

## Abstract

**Background:**

Patients infected with *Vibrio vulnificus (V. vulnificus) *show severe inflammatory responses characterised by the upregulation of proinflammatory cytokines. Macrophage migration inhibitory factor (MIF), an upstream proinflammatory regulator, increases the inflammation caused by sepsis. Whether MIF regulates responses to *V. vulnificus *infection and the actual mechanism by which *V. vulnificus *initiates these MIF-modulated proinflammatory cytokines remain unclear.

**Results:**

MIF increased inflammation during *V. vulnificus *infection *in vivo*. In *V. vulnificus*-infected mice, MIF was produced earlier than tumour necrosis factor (TNF)-α and interleukin (IL)-6 and was expressed in a time-dependent manner. ISO-1 ((S, R)-3-(4-hydroxyphenyl)-4,5-dihydro-5-isoxazole acetic acid methyl ester), a small-molecule inhibitor of MIF, significantly decreased IL-6, IL-8, and TNF-α production in a time- and dose-dependent manner in human peripheral blood cells infected with *V. vulnificus*. The induction of IL-6, IL-8, and TNF-α production by *V. vulnificus *infection was mediated via the NF-κB- and p38 MAPK-regulated pathways but not via the Akt pathway. ISO-1-treated human peripheral blood cells showed lower *V. vulnificus*-induced NF-κB activation, IL-6 mRNA expression, and IκB phosphorylation, but they did not show lower p38 MAPK activation.

**Conclusions:**

We conclude that MIF regulates *V. vulnificus*-induced IL-6 production via NF-κB activation and that p38 MAPK activation in *V. vulnificus *infection is not MIF dependent.

## Background

*Vibrio vulnificus (V. vulnificus)*, a halophilic Gram-negative bacillus, causes a serious inflammatory process involving primary septicaemia and soft tissue infections [1]. Patients with *V. vulnificus *infections have been reported in northern Europe, the United States, Australia, and Taiwan [2,3]. In the U.S., approximately 50 confirmed cases of *V. vulnificus *are reported per year, most of which occur in the Gulf Coast region. The first case was reported in Taiwan in 1985, and the number of reported infections has increased because of greater disease activity or improved recognition by clinicians [3]. Considerable data on the epidemiology of *V. vulnificus *has been obtained from Taiwan over the past two decades, and the involvement of environmental conditions, host factors, and bacterial virulence factors has led to a clearer understanding of the correlation between *V. vulnificus *infections and clinical manifestations. Numerous studies on *V. vulnificus *have investigated virulence factors, such as iron-overloading [4] and inflammation-associated cytokine production [5]. *V. vulnificus *surface structures, such as lipopolysaccharide (LPS) and capsular polysaccharides, increase cytokine production [4,5]. Further, overproduction and dysregulation of the host cytokine response to *V. vulnificus*, including tumour necrosis factor (TNF)-α, interleukin (IL)-6, and other inflammatory mediators, are critical in *V. vulnificus*-related endotoxaemic shock and lead to high mortality [6,7]. However, the mechanisms of *V. vulnificus*-initiated signal transduction for these proinflammatory cytokines remain unclear.

Macrophage migration inhibitory factor (MIF), an important proinflammatory cytokine, is a critical mediator of innate immunity and is implicated in the pathogenesis of sepsis [8,9]. Innate immune cells, including activated T cells, macrophages, and eosinophils, are the primary sites of MIF production after the host has been exposed to bacterial endotoxins and exotoxins. The released MIF modulates the expression of proinflammatory mediators, leading to early death in patients with sepsis [10-12]. In mice, the close linkage between MIF expression and Gram-negative and Gram-positive septic shock strongly suggests an intrinsic role for MIF in the innate immune response. Additionally, deleting the MIF gene or immunoneutralising MIF attenuates TNF-α production and protects against endotoxic shock [13,14]. The molecular mechanism of MIF inhibition in decreasing deleterious cytokine activity during sepsis is currently under investigation. MIF-deficient macrophages are hypo-responsive to stimulation by LPS and Gram-negative bacteria because of a defect in Toll-like receptor 4 signalling and protein expression [15]. These findings show that MIF is important in innate immunity and provide a rationale for the development of an anti-MIF strategy to treat patients with Gram-negative septic shock. The tautomerase active site of MIF has been proposed [16] as a potential target for MIF-modulating proinflammatory cytokines and might be used as a novel anti-inflammatory agent. Isoxazole acetic acid methyl ester (ISO-1), an inhibitor of MIF d-dopachrome tautomerase activity, has been shown to inhibit TNF-α secretion from LPS-treated macrophages and to protect mice from endotoxaemic [17]. The importance of ISO-1-mediated inhibition of the MIF catalytic site in the suppression of cytokine proinflammatory activity suggests that the effect of ISO-1 requires endogenous MIF.

MIF binds to the CD74-CD44 complex and induces a signalling cascade that leads to activation of downstream signalling molecules, cellular proliferation, and inhibition of apoptosis by activating the Akt pathway through the upstream proteins Src and PI3K kinase [18,19]. Nuclear factor-kappa B (NF-κB) and p38 mitogen-activated protein kinase (MAPK) are also critical intracellular signal transduction molecules involved in the pathogenesis of sepsis [20]. NF-κB, a ubiquitous transcription factor, is responsible for the transcription of a diverse range of genes involved in sepsis and is a critical regulator of genes that encode TNF-α, IL-6, chemokines, and other inducible enzymes [21,22]. As with NF-κB, p38 MAPK has been implicated as a critical mediator of the release of these proinflammatory cytokines during sepsis, and inhibiting p38 MAPK has an anti-inflammatory effect both in mice and in human endotoxaemic [20,22]. It was recently reported [23] that MIF induced the expression of TNF-related apoptosis, inducing ligand and monocyte chemoattractant protein 1 in human diabetic podocytes, and that this MIF-induced expression is also p38 MAPK dependent. We investigated the effects of MIF and its molecular actions on proinflammatory cytokine production in *V. vulnificus *infection.

## Methods

### Bacterial strains and media

A clinical isolate of encapsulated *V. vulnificus*, Chi-Mei Vv05191, was obtained from the Chi-Mei Medical Center (Tainan, Taiwan) and was prepared as previously reported [7]. A single colony was chosen and cultured at 35°C overnight in freshly prepared Mueller-Hinton broth. The bacterial suspension (400 μl) was then diluted 1:50 in Mueller-Hinton broth and incubated under the same conditions for 4 h. Bacteria were collected by centrifugation at 10,000 rpm for 10 min at 24°C. Pellets were resuspended and diluted to an inoculum with a final bacterial concentration of 1 × 10^7 ^colony-forming units (CFU)/ml (optical density: 0.22-0.25) before it was injected into mice or co-cultured with human whole blood or peripheral blood mononuclear cells (PBMCs). The inoculum concentration was confirmed by the subsequent growth of the concurrent culture on agar plates.

### Mice

Inbred BALB/c female mice (age range: 4-5 weeks; mean weight: 20 g) were purchased from the Animal Center, National Science Council, Taipei, Taiwan. They were allowed to acclimatise for 5 to 7 days in the animal research laboratory of the Chi-Mei Medical Center. Food and water were supplied ad libitum. The experimental protocol adhered to the rules of the Animal Protection Act of Taiwan and was approved by the Laboratory Animal Care and Use Committee of the Chi-Mei Medical Center.

### Preparing human cells

Buffy coats from healthy blood donors were obtained both from the Chi-Mei Medical Center and from the National Cheng Kung University Hospital. PBMCs were isolated by Ficoll-Hypaque gradient centrifugation (Pharmacia, Uppsala, Sweden) and prepared at a density of 2 × 10^6 ^cells/ml in RPMI. Blood was taken with informed consent from healthy volunteers in accordance with a protocol approved by the Human Experiment and Ethics Committees of both hospitals.

### Measuring cytokine concentration

Infected mice were killed 3, 6, 9, and 12 h after they had been intraperitoneally inoculated with the Vv05191 strain of *V. vulnificus*. Serum samples obtained from the left axillary artery were stored at -80°C until they were assayed. In the infected human-cell model, the bacterial suspensions were added to whole blood or PBMCs at multiplicities of infection (MOI, ratio of bacteria to human PBMCs/whole blood cells) from 1 to 100. At designated times, plasma or supernatants were collected for analysis using enzyme-linked immunosorbent assays (ELISA). To determine the concentrations of cytokines, ELISA kits including MIF (Chemicon International, Temecula, CA, CYT264 for mouse and R&D Systems, Minneapolis, MN, DY289 for human), IL-6 (R&D, DY406 for mouse and R&D, DY206 for human), IL-8 (R&D, DY208 for human) and TNF-α (R&D, DY410 for mouse and R&D, DY210 for human), were used according to the manufacturer's instructions.

### Inhibitors of MIF, p38 MAPK, NF-κB, and Akt

The MIF inhibitor (S, R)-3-(4-hydroxyphenyl)-4,5-dihydro-5-isoxazole acetic acid methyl ester (ISO-1) was obtained from Merck KGaA, (Darmstadt, Germany). Neutralising antibodies against human MIF and control goat IgG were purchased from R&D Systems. SB203580, pyrrolidine dithiocarbamate (PDTC), and LY294002 and wortmannin, selective inhibitors of p38 MAPK, NF-κB, and phosphoinositide 3-kinase/Akt, respectively, were purchased from Tocris Bioscience (Ellisville, MO). All drug treatments were assessed for cytotoxic effects using cytotoxicity and viability assays. Doses determined to be harmless were used.

### Western blotting

We harvested the cells and lysed them with a buffer containing 1% Triton X-100, 50 mM Tris [pH 7.5], 10 mM ethylenediamine tetraacetic acid (EDTA), 0.02% NaN_3_, and a protease inhibitor cocktail (Roche Boehringer Mannheim Diagnostics, Mannheim, Germany). After they had been freeze-thawed once, the cell lysates were centrifuged at 9,000 × *g *at 4°C for 20 min. The supernatants were then collected and boiled in sample buffer for 5 min. After they were separated using SDS-PAGE, the proteins were transferred to polyvinylidene difluoride (PVDF) membranes (Millipore, Billerica, MA), blocked at 4°C overnight in PBS-T (PBS plus 0.05% Tween-20) containing 5% skim milk, and probed with primary antibodies at 4°C overnight. After they had been washed with PBS-T, the blots were incubated with a 1:5000 dilution of horseradish peroxide (HRP)-conjugated secondary antibody at 4°C for 1 h. The protein bands were visualised using enhanced chemiluminescence (ECL) (Pierce Biotechnology Inc., Rockford, IL), and the relative signal intensity was quantified using densitometry (LabWorks analysis software; UVP, LLC, Upland, CA). The extents of IκB, Akt, and p38 MAPK activation were determined using antibodies against phosphorylation (Cell Signaling Technology, Beverly, MA).

### Electrophoretic mobility shift assays (EMSA)

Nuclear extracts from human PBMCs were prepared using a method, with minor modifications, described elsewhere [24]. The cells were washed with 2 ml of phosphate-buffered saline (PBS) and resuspended in 1 ml of PBS. The cells were then centrifuged at 2,000 × *g *for 2 min, and the supernatant was discarded. The cell pellet was incubated in 300 μl of buffer A (10 mM HEPES [pH 7.9], 1.5 mM magnesium chloride, 10 mM potassium chloride, 0.5 mM phenylmethylsulfonyl fluoride, 0.5 mM dithiothreitol, 2 μg/ml leupeptin, 10 μg/ml aprotinin, 50 mM sodium fluoride, and 1 mM sodium orthovanadate) on ice for 10 min and then gently shaken for 10 s. The pellet of the crude nuclei was collected by centrifugation at 12,000 × *g *for 10 s, resuspended in 30 μl of buffer C (20 mM HEPES [pH 7.9], 25% glycerol, 420 mM sodium chloride, 1.5 mM magnesium chloride, 0.2 mM EDTA, 0.5 mM phenylmethylsulfonyl fluoride, 0.5 mM dithiothreitol, 2 μg/ml leupeptin, 10 μg/ml aprotinin, 50 mM sodium fluoride, and 1 mM sodium orthovanadate) using a vortexer for 15 s, and then incubated on ice for 20 min. After the cells had been centrifuged at 12,000 × *g *for 2 min, the supernatant containing the nuclear proteins was collected, quantified (BCA Protein Assay Reagent; Pierce), and stored in aliquots at -70°C. The EMSA used the following oligonucleotides as probes:

NF-κ B (f): 5'-CAA ATG TGG GAT TTT CCC ATG AGT;

NF-κ B (r): 5'-GAC TCA TGG GAA AAT CCC ACA TTT G.

The forward and reverse oligonucleotides (30 pmol) were placed in 23 μl of DNA polymerase buffer (Klenow 1×; Promega), heated at 94°C for 2 min, and then annealed at room temperature for 30 min. The annealed double-stranded oligonucleotides were end-labelled using a fill-in reaction with Klenow. One unit of the Klenow and 40 μCi of [α-^32^P] dCTP (PerkinElmer) were added to the annealed oligonucleotides, and then the mixture was incubated at 30°C for 15 min. The labelled oligonucleotides were purified using G-50 columns (Sephadex; PerkinElmer Life and Analytical Sciences, Inc., Boston, MA). The DNA binding reaction was done at 4°C for 30 min in a mixture containing 3 μg of nuclear extract, 10 mM Tris-Cl [pH 7.5], 50 mM sodium chloride, 0.5 mM dithiothreitol, 0.5 mM EDTA, 1 mM magnesium chloride, 4% glycerol, 0.05 μg of poly(dI-dC)-poly (dI-dC) (PerkinElmer), and 2 × 10^4 ^cpm of ^32^P-labelled double-stranded oligonucleotides. Samples were analysed on a 4% polyacrylamide gel (acrylamide/bis-acrylamide 29:1 in 0.5× Tris borate-EDTA buffer) at 10 V/cm for 2 h. The gel was dried and analysed using quantitative autoradiography computer densitometry.

### Reverse-transcription polymerase chain reaction (RT-PCR)

The expression of mRNA was assessed using RT-PCR and quantified by real-time RT-PCR. Total cellular RNA was extracted from cells using TRIzol reagent (Invitrogen Corp., Carlsbad, CA) according to the manufacturer's instructions. RNA concentrations were quantified using a spectrophotometer (U-2000; Hitachi Koki Co., Ltd., Tokyo, Japan) at 260 nm. cDNA was prepared using reverse transcription, and PCR was done using a thermal cycler (GeneAmp PCR system 2400; PerkinElmer). Based on published sequences and sequences that we designed with Primer 3 online software http://frodo.wi.mit.edu/primer3/, we used the following oligonucleotide primers:

β-actin: sense, 5'-TGG AAT CCT GTG GCA TCC ATG AAA C-3' and

antisense, 5'-TAA AAC GCA GCT CAG TAA CAG TCC G-3';

IL-6: sense, 5'-ATG AAC TCC TTC TCC ACA AGC GC-3' and

antisense, 5'-GAA GAG CCC TCA GGC TGG ACT G-3'.

The PCR products were analysed with 1.5% agarose gel electrophoresis, stained with ethidium bromide, and then viewed with ultraviolet (UV) light using a gel camera (UVP) [25]. The levels of IL-6 mRNA were also quantified using Gene Expression Assays and 2× master mix on a StepOne Real-Time PCR system (Applied Biosystems, California, USA). Cellular RNA was extracted as described for RT-PCR and the oligonucleotide primers were as follows:

β-actin: sense, 5'-CTG GAC TTC GAG CAA GAG ATG-3' and

antisense, 5'-TGA TGG AGT TGA AGG TAG TTT CG-3';

IL-6: sense, 5'-TAC CCC CAG GAG AAG ATT CC-3' and

antisense, 5'-CAG TGC CTC TTT GCT GCT TTC-3'.

### Statistical analysis

Student's *t *test was used to analyse the data (SigmaPlot 8.0 for Windows; Systat Software, Inc., San Jose, CA). All data are represented by the means ± SD of at least three individual experiments. *P *< 0.05 was considered statistically significant.

## Results

### MIF was expressed before IL-6 and TNF-α in V. vulnificus-infected mice

**Figure 1:**
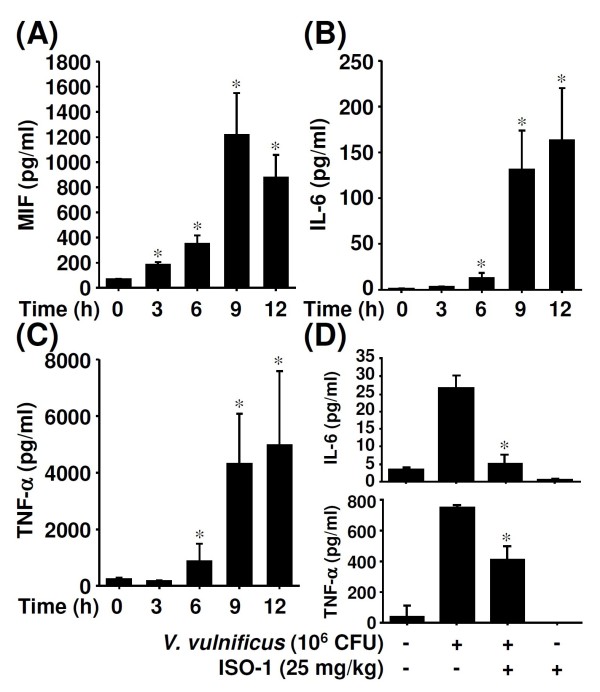
**The production of serum proinflammatory cytokines in *V. vulnificus*-infected mice**. BALB/c mice were intraperitoneally infected with *V. vulnificus *(Vv05191; 1 × 10^6 ^CFU) for the indicated time periods. ELISA was used to determine serum concentrations of MIF (A), IL-6 (B), and TNF-α (C). (D) With or without ISO-1 (25 mg/kg) pretreatment for 0.5 h, ELISA was used to determine the serum concentrations of IL-6 and TNF-α in *V. vulnificus*-infected mice at 6 h post-infection. Data are the means ± SD from three individual experiments. *P < 0.05 vs. non-infected controls or *V. vulnificus *infection only.

Blood samples from BALB/c mice were collected at 0, 1, 3, 6, 9, and 12 h after they had been infected with *V. vulnificus*, and the concentrations of the serum cytokines MIF, IL-6, and TNF-α were determined. Serum MIF peaked 9 h post-infection and then declined (Figure 1A); IL-6 and TNF-α (Figures 1B and 1C) continuously increased. Because our results showed that MIF increased earlier than IL-6 and TNF-α, we hypothesised that the early presence of serum MIF acts as an upstream enhancer or modulator of other proinflammatory cytokines, especially IL-6, during *V. vulnificus *infection. ISO-1, an MIF tautomerase inhibitor, was used to observe the effect of MIF inhibition on cytokine production in *V. vulnificus*-infected mice. Inhibition of MIF decreased *V. vulnificus*-induced production of IL-6 and TNF-α (Figure 1D).

### MIF was an upstream modulator of IL-6, IL-8, and TNF-α

**Figure 2:**
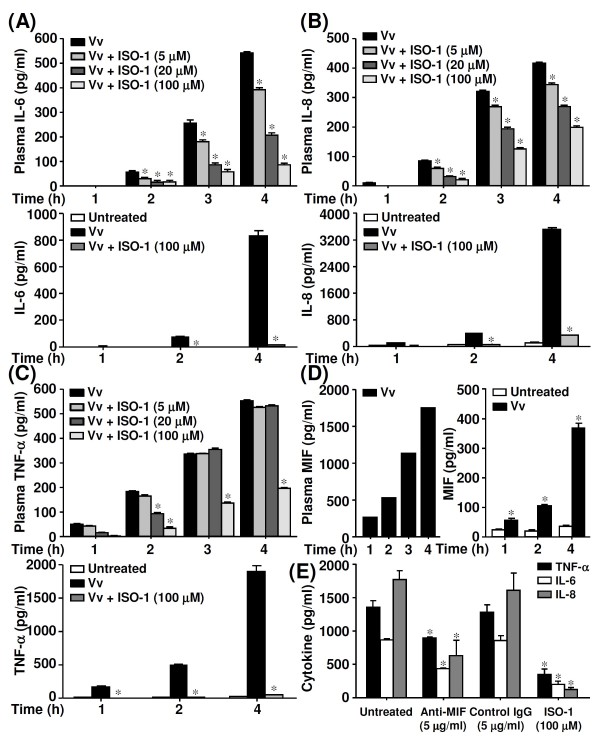
**The effects of ISO-1 on IL-6, IL-8, and TNF-α production in *V. vulnificus*-infected whole blood and isolated PBMCs**. Cells pre-treated with different doses of ISO-1 were infected with *V. vulnificus *(Vv05191; MOI = 1) for the indicated time periods. After plasma and supernatants had been collected, ELISA was used to determine IL-6 (A), IL-8 (B), TNF-α (C), and MIF (D) production. (E) With or without anti-MIF neutralising antibodies, ELISA was used to measure the concentrations of TNF-α, IL-6, and IL-8 in *V. vulnificus*-infected PBMCs at 4 h post-infection. Control goat IgG and ISO-1 were used as negative and positive controls, respectively. Data are the means ± SD from three individual experiments. *P < 0.05 vs. *V. vulnificus *infection only.

We next examined the effect of MIF inhibition on the production of the proinflammatory cytokines IL-6, IL-8, and TNF-α in *V. vulnificus*-infected human whole blood and isolated PBMCs. To inhibit MIF, PBMCs were pre-treated with ISO-1 for 30 min before they were infected with *V. vulnificus*. IL-6 (Figure 2A) and IL-8 (Figure 2B) concentrations were reduced in a time- and dose-dependent manner in the plasma of infected human whole blood and in the supernatant of infected PBMCs. However, the concentrations of TNF-α were inhibited unless the cells were pretreated with high doses of ISO-1 (Figure 2C). Moreover, different MOIs (from 10:1 to 100:1) showed the same trends of ISO-1 inhibition on IL-6, IL-8, and TNF-α (data not shown). We also confirmed that *V. vulnificus *infection induced MIF production in a time-dependent manner in infected whole blood and isolated PBMCs (Figure 2D). TNF-α, IL-6, and IL-8 were downregulated in *V. vulnificus*-infected PBMCs treated with neutralising antibodies to inhibit MIF (Figure 2E). Taken together, these results show that MIF regulated the production of IL-6, IL-8, and TNF-α during *V. vulnificus *infection.

### NF-κB and p38 MAPK signalling were essential for IL-6, IL-8, and TNF-α production

**Figure 3:**
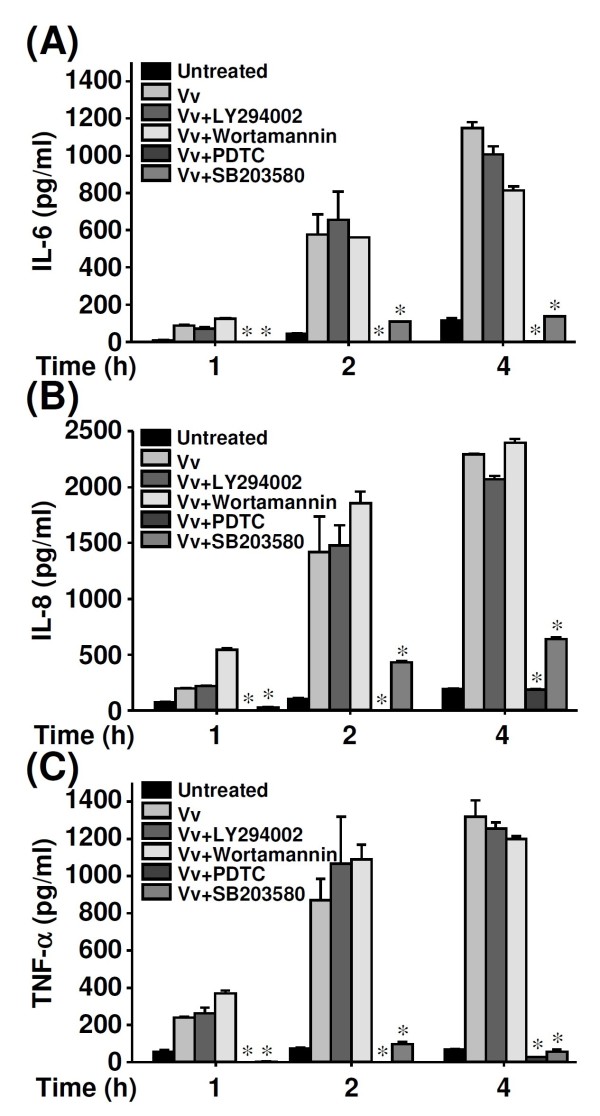
**The involvement of Akt, NF-κB, and p38 MAPK in *V. vulnificus*-induced proinflammatory activation**. Human PBMCs (2 × 10^6^/ml) were infected with *V. vulnificus *(Vv05191; MOI = 1) for the indicated time periods after they had been pre-treated for 30 min with LY294002 (100 μM), wortmannin (25 nM), PDTC (25 μM), or SB203580 (25 μM). ELISA was used to determine the concentrations of IL-6 (A), IL-8 (B), and TNF-α (C). Data are the means ± SD from three individual experiments. *P < 0.05 vs. *V. vulnificus *infection only.

We examined whether MIF-induced production of IL-6, IL-8, and TNF-α was regulated by interfering with intracellular signal transduction. The concentrations of IL-6 (Figure 3A), IL-8 (Figure 3B), and TNF-α (Figure 3C) were significantly lower in *V. vulnificus*-infected human PBMCs treated with pyrrolidine dithiocarbamate (PDTC, an NF-κB inhibitor) and SB203580 (a p38 MAPK inhibitor) but not in PBMCs treated with LY294002 or wortmannin (Akt inhibitors). These results indicate that NF-κB and p38 MAPK, but not Akt, are important for modulating IL-6, IL-8, and TNF-α during *V. vulnificus *infection.

### MIF facilitated NF-κB activation

**Figure 4:**
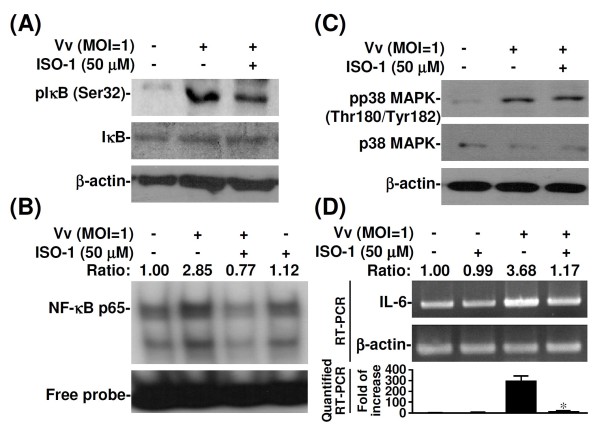
**The effects of ISO-1 on IκB, p38 MAPK activation, NF-κB DNA binding, and IL-6 expression in *V. vulnificus*-infected PBMCs**. Human PBMCs (2 × 10^6^/ml) were infected with *V. vulnificus *(Vv05191; MOI = 1) for 1 h and co-treated with ISO-1 (50 μM). Western blotting was used to measure IκB (Ser32) (A) and p38 MAPK (Thr180/Tyr182) (B) phosphorylation. β-actin was the internal control. Data are representative of three individual experiments. (C) Effects of ISO-1 on NF-κB binding. Nuclear proteins were extracted from cells after they had been infected with *V. vulnificus *and co-treated with or without ISO-1. An EMSA assay was used to analyse *V. vulnificus*-infected cells co-treated with ISO-1 for 15 min and to analyse nuclear proteins (3 μg). Lane 1: Control; Lane 2: *V. vulnificus*-infected cells; Lane 3: *V. vulnificus*-infected cells + ISO-1; Lane 4: ISO-1 only; (D) Effects of ISO-1 on IL-6 mRNA expression. Human PBMCs (2 × 10^6^/ml) were infected with *V. vulnificus *(Vv05191; MOI = 1) for 1 h and co-treated with ISO-1 (50 μM). The ratio represents the quantitative densitometry analysis of bands compared with untreated controls. β-actin was the internal control. For real-time RT-PCR analysis, the data is shown as a normalized ratio. Data are representative of three individual experiments. Data are the means ± SD from three individual experiments. *P < 0.05 vs. *V. vulnificus *infection only.

Both NF-κB and p38 MAPK are essential mediators of the response of inflammatory cytokines to either Gram-negative or Gram-positive bacterial sepsis [20-22,26,27]. We next examined whether MIF modulated NF-κB- or p38 MAPK-dependent signalling transduction during *V. vulnificus*-induced proinflammatory activation. Western blotting showed that the phosphorylation of IκB was lower in ISO-1-treated *V. vulnificus*-infected human PBMCs (Figure 4A); however, p38 MAPK phosphorylation was not lower (Figure 4B). These results suggested that MIF is an upstream modulator of NF-κB-dependent signalling for IL-6, IL-8, and TNF-α production during *V. vulnificus *infection.

### NF-κB was essential for the transcriptional activation of MIF-induced IL-6 expression

Because NF-κB is an important transcription factor for IL-6 expression and binds to its response element on the IL-6 promoter [20,21,27], we examined whether increased NF-κB binding activity upregulates IL-6 in *V. vulnificus*-infected human PBMCs. We used EMSA with probes containing the putative NF-κB binding site on the IL-6 promoter to measure NF-κB binding activity. After the PBMCs had been infected with *V. vulnificus*, NF-κB binding was higher in control cells than in PBMCs treated with ISO-1 (Figure 4C, lanes 2, 3). RT-PCR and real-time RT-PCR showed that IL-6 mRNA levels were lower in *V. vulnificus*-infected PBMCs treated with ISO-1 than in those not treated with ISO-1 (Figure 4D). Taken together, these results suggest that MIF is crucial upregulator of IL-6 at the transcription level in *V. vulnificus*-infected cells and that this upregulation is the result of NF-κB activation.

## Discussion

In this study, we showed that MIF regulated the production of TNF-α, IL-6, and IL-8 in *V. vulnificus*-infected human PBMCs, which is consistent with the findings of other studies [8,9]. High serum levels of IL-6, a crucial immunoregulator in patients with early-phase Gram-negative sepsis, predict poor outcomes for patients with sepsis [28,29]. IL-6 increases at early time points in patients with severe sepsis and septic shock [12,30]. IL-8 also is induced in human intestinal epithelial cells infected with *V. vulnificus*, and IL-8 mRNA expression increases as the result of NF-κB activation [31]. In the present study, we provide evidence that MIF upregulates IL-6, IL-8, and TNF-α in cells infected with *V. vulnificus*. We hypothesise that MIF is an initial proinflammatory mediator during *V. vulnificus *infection, which is consistent with previous reports [12,14] on patients with sepsis and endotoxaemic models.

We showed that *V. vulnificus *infection significantly increased IL-6 mRNA expression and NF-κB binding in human PBMCs. We also showed that *V. vulnificus *infection increased IL-6 gene expression via NF-κB activation in human PBMCs and that ISO-1, a small molecule inhibitor of MIF, downregulated the transcription of IL-6 and NF-κB activation. While ISO-1 has a different effect on Toll-like receptor 4-induced inflammatory responses [32], our findings indicate that IL-6 gene expression is regulated by MIF activation of NF-κB. Although *V. vulnificus *infection induced MIF-regulated proinflammatory cytokines, only high-dose treatments of ISO-1 inhibited TNF-α production, which indicated that an MIF-independent signalling pathway might exist for regulating TNF-α during *V. vulnificus *infection.

In the present study, we found that the p38 MAPK and NF-κB pathways were the major signalling pathways through which cytokine release was induced in human PBMCs infected with *V. vulnificus*. Furthermore, a recent study [26] reported that p38 MAPK was essential for group B streptococcus-induced MyD88-dependent induction of TNF-α production but was unnecessary for NF-κB activation [26]. However, based on our findings, it appears that *V. vulnificus*-induced IL-6 production is facilitated by MIF via NF-κB activation but not by p38 MAPK. The different responses and their molecular mechanisms need further investigation.

Inhibiting MIF with ISO-1 downregulated the phosphorylation of IκB and partially inhibited NF-κB binding activity and the mRNA expression of IL-6, which suggested that MIF is an important upregulator of IL-6 production via NF-κB activation. Although the ISO-1-mediated downregulation of NF-κB binding and IL-6 mRNA expression was less significant than the downregulation of IL-6 production, the discrepancy between the upregulation of transcription and protein production suggests that these processes are controlled by different mechanisms. In our preliminary studies, we found a significant decrease in protein production after ISO-1 treatment in both the intracellular and extracellular phases. However, the mechanism that ISO-1 uses to inhibit intracellular protein production is unknown.

## Conclusion

We have shown that MIF significantly upregulated IL-6 production by facilitating NF-κB activation in human PBMCs infected with *V. vulnificus*. These data may help in the development of strategies to target MIF with specific inhibitors or neutralising antibodies when treating *V. vulnificus *infection.

## List of abbreviations

ISO: isoxazole acetic acid methyl ester; MIF: macrophage migration inhibitory factor; TNF-α: tumour necrosis factor alpha; IL: interleukin; NF-κB: nuclear factor-kappa B; MAPK: mitogen-activated protein kinase; MOI: multiplicity of infection; CFU: colony-forming units; PBMCs: peripheral blood mononuclear cells; PBS: phosphate-buffered saline; SDS-PAGE: sodium dodecyl sulphate polyacrylamide gel electrophoresis; ELISA: enzyme-linked immunosorbent assay; EMSA: electrophoretic mobility shift assay; RT-PCR: reverse transcription-polymerase chain reaction; mRNA: messenger RNA.

## Competing interests

The authors declare that they have no competing interests.

## Authors' contributions

CCC (Chuang) and CFL conceived of and designed the study. CCC drafted the manuscript. WTC, CCC (Chen), PCC, CYW, and PCT performed the immunoassays and biochemical analyses. LIH participated in design of the study and helped revise the manuscript. YCC and AMH supervised the study. All authors read and approved the final manuscript.
